# Multimodality Treatment May Improve the Survival Rate of Patients with Metastatic Nasopharyngeal Carcinoma with Good Performance Status

**DOI:** 10.1371/journal.pone.0146771

**Published:** 2016-01-12

**Authors:** Wei Zheng, Jingfeng Zong, Chaobin Huang, Juhui Chen, Junxin Wu, Chuanben Chen, Shaojun Lin, Jianji Pan

**Affiliations:** 1 Department of Radiation Oncology, Fujian Provincial Cancer Hospital, Provincial Clinical College of Fujian Medical University, Fuzhou, Fujian 350014, People’s Republic of China; 2 Fujian Provincial Key Laboratory of Translational Cancer Medicine (Fujian Provincial Cancer Hospital, Fujian Medical University Union Hospital), Fuzhou, Fujian 350014, People’s Republic of China; Seoul National University, REPUBLIC OF KOREA

## Abstract

The aim of the present study was to evaluate the benefit of chemotherapy, combined with palliative radiotherapy (PRT) and other local treatments to the metastatic sites, for patients with metastatic nasopharyngeal carcinoma (NPC) who had a performance status 0–2. We conducted a retrospective review of available data from 197 biopsy-proven NPC patients who developed metastasis after their initial definitive treatment. These patients were grouped into three categories according to the different treatment paths that were followed: the best supportive care (64 patients), chemotherapy alone (55 patients), and multimodality treatment with chemotherapy combined with PRT and other local treatments to metastatic sites (78 patients). The 2-year metastatic survival rate of patients in the multimodality treatment group was 57.7%, which was significantly better than that of the patients in both the chemotherapy alone group and the best supportive care group (32.7% and 1.6%, respectively). The independent significant factors affecting survival were the disease-free interval prior to the detection of metastatic disease, the number of metastases, the number of chemotherapy cycles and the biological effective dose of PRT. In conclusion, multimodality treatment may improve survival of select patients with recurrent NPC with distant metastases.

## Introduction

Chemoradiotherapy is the mainstay treatment for nasopharyngeal carcinoma (NPC) [1]. Despite the ability of chemoradiotherapy using intensity modulated radiotherapy (IMRT) technology to achieve locoregional control in over 90% of patients with non-metastatic NPC at presentation, the distant failure rate following initial definitive IMRT is as high as 14.1% [2] and is the foremost reason for treatment failure for patients with NPC [3].

Chemotherapy is considered an effective treatment method for patients who develop distant metastasis, due to the high objective response rates and a substantial proportion of durable complete responses [4]. Several small-sample studies have indicated that patients may achieve long-term disease-free survival if given aggressive chemotherapy [5,6]. However, the reported median survival times were only 11–22 months, and patients seldom survived longer than three years [4].

Some recently reported studies have demonstrated that combined modality therapy, including chemotherapy and palliative radiotherapy (PRT) of metastatic lesions, offer a survival advantage for patients with metastatic NPC in addition to offering an improved quality of life [7,8]. Confirmation of those results would help change the current clinical treatment strategy for metastatic NPC.

In the present study, the retrospective data from metastatic NPC patients with a good performance status after the initial radical treatment were collected, the survival rates were evaluated, and the factors affecting the patient survival were analyzed to assess the benefit of chemotherapy combined with PRT and other local treatments to metastatic sites.

## Materials and Methods

### Patient characteristics

A total of 302 pathologically confirmed NPC patients with distant metastasis after primary radical treatment were treated at Fujian Provincial Cancer Hospital (South China) from Jan 1, 2001 to Dec 31, 2010. We excluded individuals from the study who had received their initial treatment at other hospitals (47 patients). We also excluded individuals who had local and/or regional relapse with the diagnosis of distant metastasis (40 patients), those considered ineligible for systemic chemotherapy due to medical conditions (six patients), those with other prior malignancies (four patients) and those with a poor Eastern Cooperative Oncology Group (ECOG) performance status of 3 or more (eight patients). The remaining 197 patients were included in the study. Our retrospective analysis was approved by the Fujian Provincial Cancer Hospital Institutional Review Board. Although patient consent was not specifically obtained for this retrospective review, all information had been anonymized and de-identified prior to its analysis.

Histologically, all 197 enrolled patients had World Health Organization type III disease (undifferentiated non-keratinizing carcinoma). The group comprised 164 male patients and 33 female patients, with a male-female ratio of 4.97:1. All patients were initially treated with definitive external beam radiotherapy. A total of 167 patients (84.8%) received platinum-based chemotherapy as part of their initial treatment. At the time of diagnosis of metastasis, the median age was 48 years (range, 14–76 years). Bone, pulmonary, hepatic and distant lymph nodes metastases were diagnosed in 55, 42, 34 and 8 patients, respectively, whereas 58 patients presented with multiple organ metastases.

### Pre-treatment Evaluation and Definition of Metastases

All patients received a pretreatment evaluation following the diagnosis of a distant metastasis, consisting of a complete medical history and physical examination, flexible fiberoptic endoscopy examination, standard laboratory tests, electrocardiogram, chest X-ray, computed tomography (CT) or magnetic resonance imaging (MRI) scan of the head and neck, Technetium-99m bone scan, and ultrasound of the liver and abdomen. Abdominal and thoracic CT scans, and MRI of the liver or bone were performed when clinically indicated.

The diagnosis of metastasis was made in accordance with the following: (1) metastatic lesions confirmed by surgery or biopsy pathology, (2) CT or 18F-FDG PET-CT prompted multimetastasis, (3) two or more than two types of imaging examination confirming a single metastatic lesion [9]. The detection of one solitary or two metastatic lesions was defined as oligometastasis; and the detection of three or more metastatic lesions was defined as multimetastasis.

Oligometastasis was present in 24.9% of patients (49/197). Of the 49 patients, a total of 37 patients (14 patients with pulmonary lesions, 16 with hepatic lesions, four with lesions in distant lymph nodes outside the neck and head, and three with in both lung and bone) were confirmed by pathology. Only 12 patients with metastasis in bone alone were diagnosed based on at least two types of imaging examinations (ECT or MRI or CT) and excluded other primary neoplasms after thorough physical and imaging evaluation that were accepted as the criteria for diagnose of bone metastasis [10].

### Treatments

All 197 patients were allocated to one of three categories of treatment: to receive the best supportive care, chemotherapy alone or multimodality treatment. Sixty-four patients were to receive best supportive care, of which six were referred for palliative radiotherapy for their symptomatic metastasis. Fifty-five patients received chemotherapy alone. Multimodality treatment was carried out for 78 patients, among whom three underwent surgery (one case had resection of lung metastasis and two cases of hepatic metastases), two cases received alcohol injection therapy for liver metastasis and 73 cases were prescribed palliative radiotherapy to metastatic sites. Among the patients who received radiotherapy, three were treated with IMRT for their pulmonary foci, six were treated with three-dimensional conformal radiotherapy (3D-CRT) (two cases in the axillary lymph nodes and four for pulmonary metastasis), and 64 were treated by 2D-CRT. The radiotherapy doses ranged between 30–70 Gy with 2–4 Gy/fraction. For the convenience of analysis, the biological effective dose (BED) was used for comparison. The biological effective dose (BED) was used to calculate the different radiation schedules [11], assuming the α/β ratio of 10 Gy for tumor cell killing.

**Fig 1:**
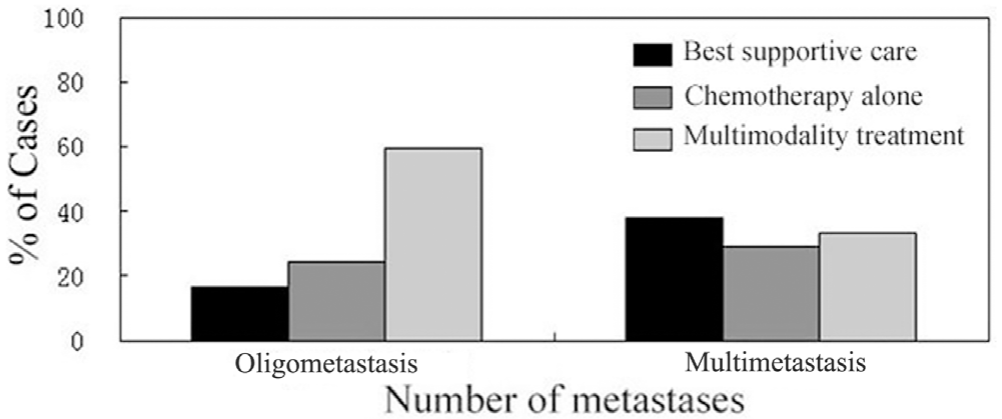
Distribution of treatment modalities and the state of metastatic lesions (oligometastasis or multimetastasis).

The patient characteristics according to different treatment modalities are listed in Table 1. Patients with oligometastasis were significantly more likely to receive multimodality treatment compared to patients with multimetastasis (Fig 1, *P* = 0.003). There were no significant differences in the other clinical characteristics between the three groups of patients (Table 1).

**Table 1:** Clinical characteristics of patients with NPC developing distant metastasis.

Characteristic	BSC	Ch-A	Multi-T	χ^2^	P value
**Gender**				1.866	0.39
**Male**	53	43	68		
**Female**	11	12	10		
**Age (years)**				0.847	0.655
**≤40**	19	20	23		
**>40**	45	35	55		
**ECOG**				5.113	0.276
**0**	20	23	25		
**1**	39	23	43		
**2**	5	9	10		
**Metastases number**				11.84	0.003
**Oligometastasis**	8	12	29		
**Multimetastasis**	56	43	49		
Primary chemotherapy*				1.415	0.493
**No**	7	9	14		
**Yes**	57	46	64		
**Primary T stage**				0.268	0.875
**T1-2**	20	17	27		
**T3-4**	44	38	51		
**Primary N stage**				1.203	0.548
**N0-1**	24	26	34		
**N2-3**	40	29	44		
**Primary clinical stage**				1.745	0.783
**Stage II**	10	5	13		
**Stage III**	31	28	38		
**Stage IV**	23	22	27		
**Total (n)**	64	55	78		

ECOG: Eastern Cooperative Oncology Group; BSC: Best supportive care; Ch-A: Chemotherapy alone; Multi-T: Multimodality treatment.

*: Chemotherapy as part of initial treatment of nasopharyngeal carcinoma

For the 133 patients who chose chemotherapy, the median number of treatment cycles was three (range, 1–18). Ninety-four patients received at least three cycles of chemotherapy. Fifty-six patients received a TP regimen (paclitaxel and cisplatin), 46 received a GP regimen (gemcitabine and cisplatin), and the other 31 accepted a PF regimen (cisplatin and 5-FU). Among patients where first-line chemotherapy failed, 12 received oxaliplatin-based chemotherapy, 12 were prescribed other cisplatin-based chemotherapy, and one was switched to capecitabine monotherapy. The average number of cycles administered was 4.0±3.7 cycles for the chemotherapy alone group and 4.5±2.8 cycles for the multimodality treatment group (t = 0.856, *P* = 0.394).

### Follow-up and statistical analysis

All patient outcomes were evaluated in December 2014. The metastatic survival time was calculated from the date of diagnosis of distant metastasis to the date of death or the last follow-up. The disease-free interval (DFI) was calculated from the completion date of initial head and neck radical radiotherapy to the date of diagnosis of the first distant metastasis.

The survival data were analyzed with SPSS software, version 18.0 (SPSS, Inc., Chicago, IL, USA). Survival curves were created using the Kaplan-Meier method and compared with the log-rank test. A multivariate analysis by gender, age, ECOG score, primary T/N stage, DFI, number of metastases, number of chemotherapy course and BED to metastatic foci was performed using the Cox proportional hazards model. In cases where patients underwent more than one course of palliative radiotherapy for metastatic lesions, the BED was defined depending on the initial treatment course. The criterion for statistical significance was set at α = 0.05 and P-values were based on two-sided tests.

## Results

### Characteristics of metastasis

The median DFI of all patients was 14 months (range, 1–112 months). Most instances of metastatic disease (73%) developed during the first two years after primary radiotherapy with 20 (10.2%) and 47 (23.9%) patients affected during the first three and six months, respectively. Only 12.2% of the metastatic tumors were detected after three years.

### Survival time

The median follow-up time was 36 months (range, 3–183 months). At the last follow-up, 15 patients were alive and 182 cases succumbed as a result of their disease. The median metastatic survival time in all patients was 14 months (range 1–132 months), and the 1-, 2- and 3-year metastatic survival rates were 54.8%, 32.5% and 20.3%, respectively.

**Fig 2:**
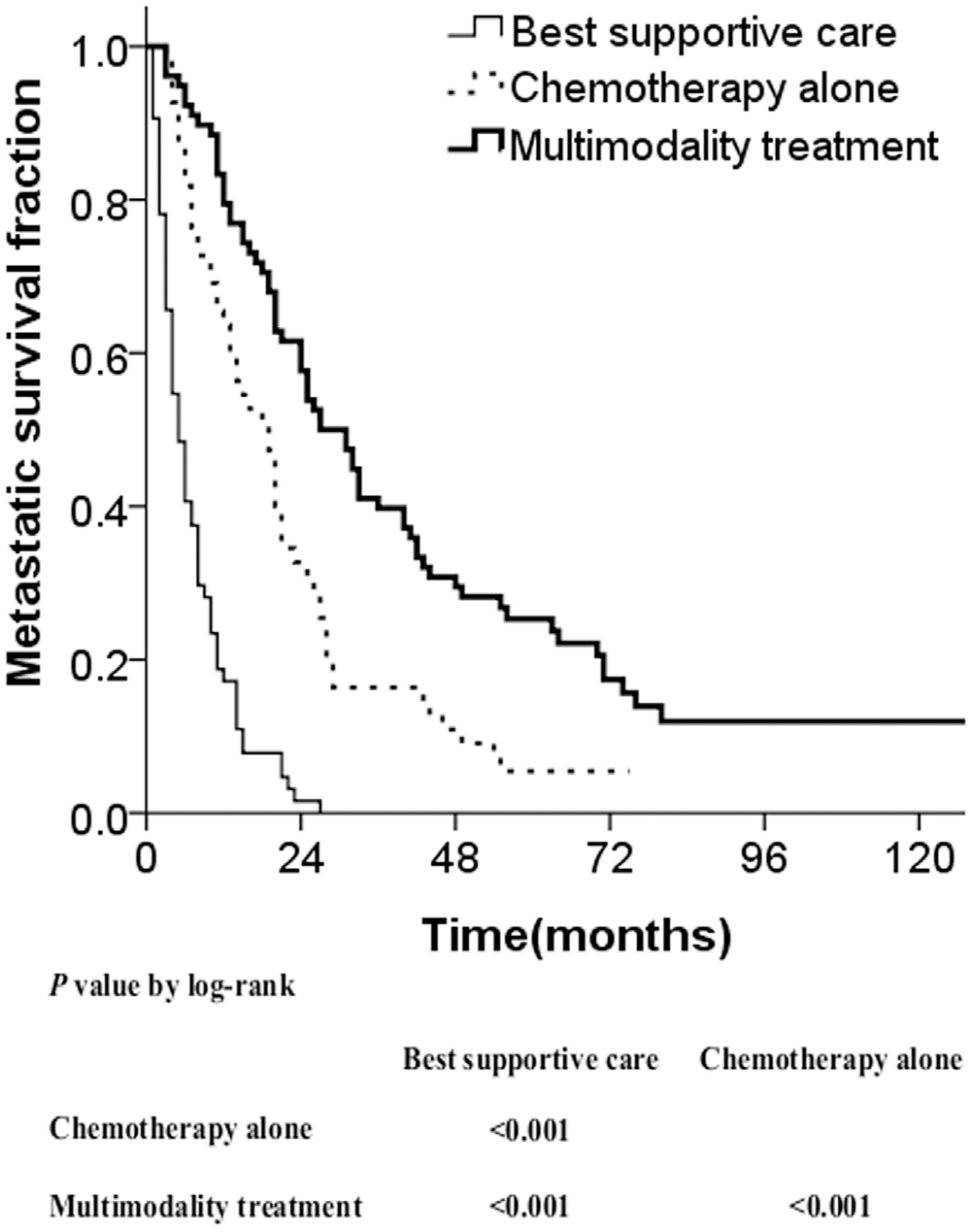
Metastatic survival curves stratified by treatment modality.

The median metastatic survival time for patients having the best supportive care was five months (range, 1–27 months), with a 2-year metastatic survival rate of 1.6%; for patients receiving chemotherapy alone it was 19 months (range, 3–75 months) with a rate of 32.7%; and for the multimodality treatment group it was 29 months (range, 5–55 months) with a rate of 57.7%. The metastatic survival with chemotherapy alone was superior to survival with best supportive therapy (χ^2^ = 40.913, *P* <0.001), whereas it was inferior to survival with multimodality treatment (χ^2^ = 12.968, *P* <0.001) (Fig 2).

### Univariate and multivariate analyses

The metastatic survival rate was higher for the following subgroups among these variables in the entire population: survival of patients with DFI ≥1 year was better compared to <1 year, BED of PRT to metastatic foci ≥50 Gy produced a better survival compared to <50 Gy, oligometastasis was better compared to multimetastasis; and ≥4 cycles of chemotherapy was associated with better survival compared to <4 cycles (Table 2). Multivariate analysis showed that the DFI, the number of metastases, the number of chemotherapy cycles and BED were independent prognostic factors (Table 3).

**Table 2:** Univariate analysis of clinical variables for metastatic survival rate.

Clinical variables	HR	95% CI for HR	*P* value
**Age(≤40 vs. >40 years)**	1.25	0.906–1.724	0.174
**Gender(female vs. male)**	1.058	0.715–1.566	0.776
**Primary T stage (T1-2 vs. T3-4)**	0.981	0.885–1.088	0.717
**Primary N stage (N0-1 vs. N2-3)**	1.061	0.916–1.228	0.428
**DFI (<1 year vs. ≥1 year)**	0.692	0.516–0.927	0.014
**ECOG performance status(0–1 vs. 2)**	1.121	0.900–1.397	0.307
**Metastases detected (Oligo vs. Multi)**	1.596	1.321–1.929	<0.001
**BED (<50 Gy vs. ≥50 Gy)**	0.426	0.280–0.646	<0.001
Chemotherapy cycles* **(<4 vs.≥4)**	0.566	0.414–0.773	<0.001

HR: hazard ratio; CI: confidence interval; DFI: disease-free interval; BED: biological effective dose of palliative radiotherapy to metastatic site.

*: chemotherapy cycles after metastatic disease

**Table 3:** Multivariate analysis of clinical variables for metastatic survival rate.

Variable	HR	95% CI for HR	*P* value
**DFI (<1 year vs. ≥1 year)**	0.697	0.517–0.94	0.018
**Metastases detected (Oligo vs. Multi)**	1.578	1.302–1.912	<0.001
**Chemotherapy cycles (<4 vs. ≥4)**	0.576	0.419–0.791	<0.001
**BED (<50 Gy vs. ≥50 Gy)**	0.487	0.319–0.744	<0.001

HR: hazard ratio; CI: confidence interval; DFI: disease-free interval; BED: biological effective dose of palliative radiotherapy to metastatic site.

### Stratified analysis

We performed stratified analyses according to the DFI and the number of metastases detected to identify which subgroups of metastatic NPC patients benefited from the multimodality treatment.

**Fig 3:**
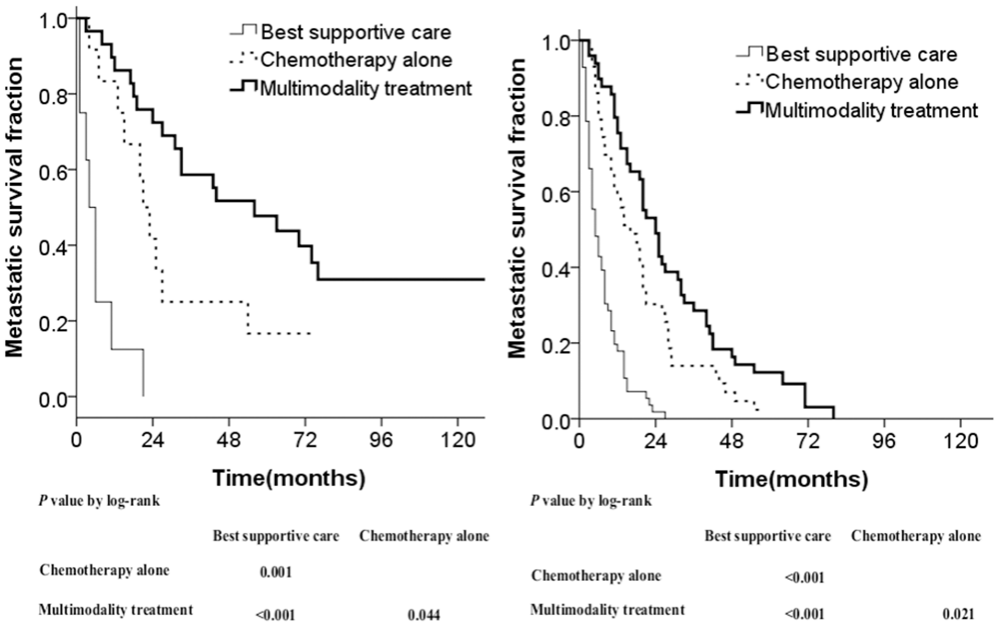
Metastatic survival curves stratified by treatment modalities in different number of metastases: oligometastasis (A) and multimetastasis (B).

**Fig 4:**
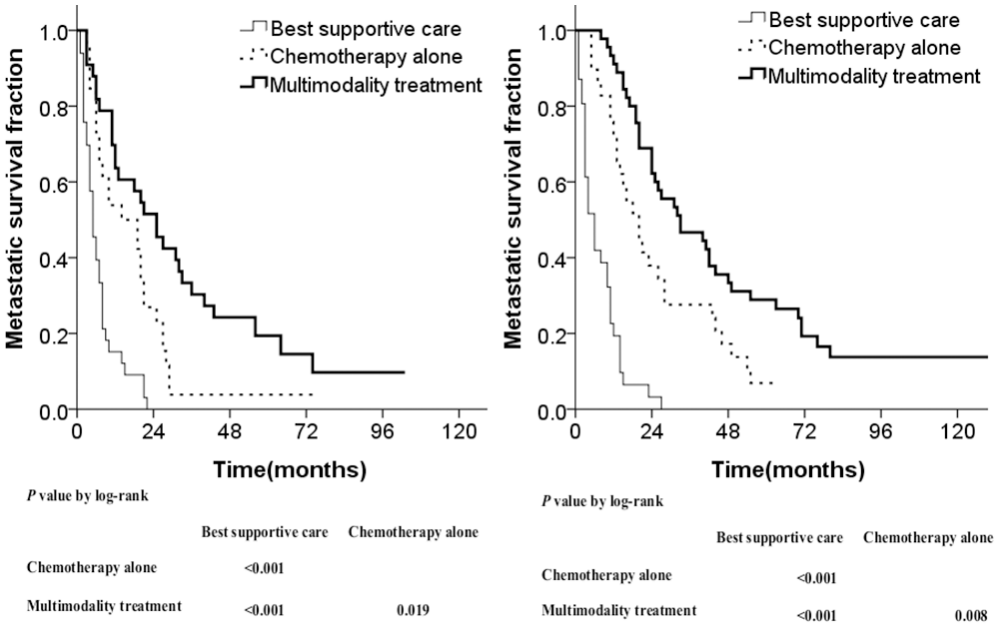
Metastatic survival curves stratified by treatment modalities in different disease-free intervals (DFI): DFI <1 year (A) and DFI >1 year (B).

The results showed that patients in the multimodality treatment group presented with the best survival compared to those in the chemotherapy alone or best supportive care group among both oligometastasis and multimetastasis patients (Fig 3), with improved outcomes in patients with fewer metastases. Treatment modalities could also stratify the outcome of patients with a DFI ≥1 year compared with <1 year (Fig 4). The survival curves of these three treatment groups showed good segregation.

## Discussion

Our study has confirmed that multimetastasis and a short metastasis-free interval are capable of predicting a significantly shorter survival time, as has been described by other published studies [12,13,6]. Additionally, 47 patients (23.9%) were found to have distant metastasis during the first six months following primary radiotherapy in this study. Early metastatic spread or micro-metastatic disease may already have occurred at the time of initial diagnosis, but had remained undetected by the conventional clinical work-up in these patients with such a short DFI. Our study cohort includes individuals who had been examined when PET-CT was not part of our staging preparation. In addition to PET-CT, it has also been reported that screening for plasma Epstein-Barr virus DNA can also detect early distant metastasis for untreated NPC [14].

Chemotherapy has often been used to relieve the symptoms of patients with distant organ metastasis. To date, no study has determined whether chemotherapy could really improve the survival rate for metastatic NPC compared to the best supportive care. Our retrospective study showed that patients who received chemotherapy had a significantly improved 2-year survival rate compared with those who declined chemotherapy (32.7% vs. 1.6%, *P* <0.001). Our results also showed that patients who received ≥4 cycles of chemotherapy had a better prognosis compared with those who received <4 cycles (*P* <0.001) and these results are in accordance with those of Jin, et al. [15]. Therefore, four to six cycles of chemotherapy are recommended for metastatic NPC, unless contraindicated [6,5].

PRT, directed at palliation of symptoms and improving the quality of life, is one of the most common therapeutic options for treating metastatic lesions, which is often delivered according to the attending doctor's experience. Other local treatment methods for metastatic foci include surgical resection, or interventional treatment for liver metastasis such as transcatheter arterial chemo-embolization, percutaneous ethanol injection, and radiofrequency ablation. Whether these palliative local treatments, when combined with systemic chemotherapy, can also prolong the life of patients following metastatic NPC has long been questioned. In our study, 133 patients received chemotherapy. Among these, only 78 received active additional local treatment to metastatic sites because of the variable extent of the metastatic lesion or differing perspectives of the attending physicians. The results showed that the metastatic survival rate of patients in the multimodality treatment group was significantly improved compared to the patients who received chemotherapy alone (Fig 2).

Patients with oligometastasis were more prone to accept multimodality treatment compared with those having multimetastasis, therefore, we calculated the survival times of the three different treatment groups using the number of metastases as a stratification factor. This showed that regardless of whether patients had oligometastasis or multimetastasis, the best supportive care group fared significantly worse compared to either of the other two treatment groups, and the chemotherapy alone group fared significantly worse than the multimodality treatment group (Figs 3 and 4).

No optimum radiation dose or fractionation schemes for PRT had been determined. Our data revealed that patients receiving a BED of ≥50 Gy experienced an improved prognosis compared with those receiving <50 Gy (*P* <0.001). Therefore, to promote survival as well as palliate the symptoms of patients with metastatic NPC, at least 50 Gy of a BED dose maybe considered for the administration of local radiation to metastatic foci.

Currently, no standard treatment guidelines exist for metastatic NPCs [4]. Patients with distant organ metastasis are usually treated as candidates for all types of clinical trials to evaluate the outcome of varied chemotherapy regimens or novel targeted drugs. Local treatments for metastatic foci, except for low-dose PRT to no-target bone metastases, are mostly avoided to prevent a treatment bias between different randomized groups [16]. We are concerned that this type of management may not be in the best interest of some patients who may obtain long-term survival benefits from combined systemic chemotherapy with other consolidated local treatment limited to metastases [17]. Based on the results of our study, we recommend that multimodality treatment consisting of local radiotherapy to metastatic foci and systemic chemotherapy should be included in a protocol for the treatment of patients with NPC and distant metastasis.

The major drawback of our study is that although the multivariate analysis might assist to decrease the uncertainty of the results to some extent, the retrospective nature may still involve the issue of patient selection bias. Secondly, most (64/87, 82%) of the patients received conventional radiation therapy, while with the development of stereotactic body radiotherapy, the treatment to the multimetastatic lesions, especial to the pulmonary oligometastases, might achieve better results [18]. Thirdly, limitations of the number of cases, and the efficacy of multimodality treatment in different metastatic sites were not analyzed in the current study. In addition, patients were excluded from the study if they had sophisticated treatment of distant metastases concomitant with local recurrence. A separate evaluation of this group would be of future interest.

In conclusion, our study supports local treatment, including palliative radiotherapy combined with systemic chemotherapy, being effective for achieving a longer survival time in some patients with metastatic NPC when compared with patients receiving chemotherapy alone.

## Supporting Information

S1 Appendix Clinical data of enrolled patients. (XLSX)
